# Genomic Analysis of Cardiac Surgery–Associated *Mycobacterium chimaera* Infections, United States 

**DOI:** 10.3201/eid2503.181282

**Published:** 2019-03

**Authors:** Nabeeh A. Hasan, L. Elaine Epperson, Adrian Lawsin, Rachael R. Rodger, Kiran M. Perkins, Alison Laufer Halpin, K. Allison Perry, Heather Moulton-Meissner, Daniel J. Diekema, Matthew B. Crist, Joseph F. Perz, Max Salfinger, Charles L. Daley, Michael Strong

**Affiliations:** National Jewish Health, Denver, Colorado, USA (N.A. Hasan, L.E. Epperson, R.R. Rodger, M. Salfinger, C.L Daley, M. Strong);; Centers for Disease Control and Prevention, Atlanta, Georgia, USA (A. Lawsin, K.M. Perkins, A.L. Halpin, K.A. Perry, H. Moulton-Meissner, M.B. Crist, J.F. Perz);; University of Iowa, Iowa City, Iowa, USA (D.J. Diekema)

**Keywords:** Mycobacterium chimaera, outbreak, genomics, nosocomial infection, cardiac surgery, heater–cooler unit, nontuberculous mycobacteria, bacteria, United States, tuberculosis and other mycobacteria

## Abstract

A surgical heater–cooler unit has been implicated as the source for *Mycobacterium chimaera* infections among cardiac surgery patients in several countries. We isolated *M. chimaera* from heater–cooler units and patient infections in the United States. Whole-genome sequencing corroborated a risk for these units acting as a reservoir for this pathogen.

*Mycobacterium chimaera* is a species in the *Mycobacterium avium* complex (MAC) (*1*). MAC is the most frequently reported cause of nontuberculous mycobacterium (NTM) infection in the United States, although disseminated *M. chimaera* infections are relatively rare (*2*). In 2012, investigators in Switzerland found that some patients with disseminated *M. chimaera* infections had undergone open-chest cardiac surgeries, during which they were exposed to heater–cooler units (HCUs) (*3*). These devices, Stöckert 3T Heater–Cooler Units (LivaNova PLC, https://www.livanova.com; formerly Sorin Group Deutschland GmbH), manufactured in Germany, were unknowingly contaminated with *M. chimaera* (*4*,*5*). In the same year, a Pennsylvania hospital identified a cluster of invasive *M. chimaera* infections among open-chest cardiac surgery patients exposed to LivaNova 3T HCUs contaminated with *M. chimaera* (*6*), which prompted notification of ≈1,300 patients with exposure to these units (*7*). Additional cases of disseminated *M. chimaera* infection among cardiac surgery patients have emerged worldwide, with evidence implicating bioaerosols produced by contaminated LivaNova 3T HCUs as the source of post–cardiac surgery *M. chimaera* infections (*8*,*9*). We report the relationships among HCU-associated isolates from patients and LivaNova 3T HCUs in the United States and their context among the global outbreak.

## The Study

During 2015–2016, we collected NTM isolates from 3T HCU water (n = 38 isolates) and suspected patient cases (n = 24 isolates) from 8 US locations. We identified isolates and conducted high-throughput whole-genome sequencing using the Illumina Miseq system (https://www.illumina.com). We selected Pennsylvania isolate 2015-2271 (USA_PA_PAT_9) for Pacific Biosciences (https://www.pacb.com) single-molecule real-time sequencing (*10*). We downloaded publicly available *M. chimaera* genomes from isolates collected in Australia, Denmark, Italy, New Zealand, the United Kingdom, and Switzerland from the National Center for Biotechnology Information (NCBI) Sequence Read Archive (SRA). We included Zürich CHE_HCU_1 isolate as a representative of the genotype isolated from HCUs, patients, and manufacturing sites in Europe (*9*). For each isolate, we mapped the sequence reads to the *M. chimaera* strain CDC 2015-22-71 reference genome (GenBank accession no. NZ_CP019221.1) to detect single-nucleotide polymorphisms (SNPs) (Appendix).

We reconstructed phylogenetic relationships among *M. chimaera* isolates collected from post–cardiac surgery patients and HCUs in 8 locations across the United States, as well as HCU-associated strains from Australia, New Zealand, and Europe (Table; Appendix Figure 1). We compared all HCU-associated isolates with 7 *M. chimaera* respiratory isolates obtained from US patients with no history of cardiac surgery. We identified 18,190 SNPs in the 3.82-Mb core genome (62.8% of the reference genome) among 126 *M. chimaera* isolates.

**Table:** *Mycobacteria chimaera* isolated from HCUs, suspected patient case(s), and non–HCU-associated *M. chimaera* isolates in Australia, Europe, New Zealand, and the United States*

Location	No. isolates	No. clinical	No. HCU	Status	Genotypes/location	No. HCU1 genotypes (%)	NCBI BioProject no.	Reference
Iowa 1	9	3	6	HCU	1	9 (100)	PRJNA345021	(*11*); this study
Iowa 2	3	0	3	HCU	1	3 (100)	PRJNA345021	This study
Kentucky	1	0	1	HCU	1	1 (100)	PRJNA345021	This study
Massachusetts 1	4	0	4	HCU	1	4 (100)	PRJNA345021	This study
Michigan	17	8	9	HCU	1	17 (100)	PRJNA345021	This study
Minnesota	1	1	0	HCU	1	1 (100)	PRJNA345021	This study
Pennsylvania 1†	25	10	15	HCU	2	23 (92)	PRJNA344472	(*11*); this study
Virginia	2	2	0	HCU	1	2 (100)	PRJNA345021	This study
Australia 1	6	0	6	HCU	1	6 (100)	PRJEB15375	(*12*)
Australia 2	13	1	12	HCU	3	11 (84.6)	PRJEB15375	(*12*)
Australia 3	7	2	5	HCU	3	5 (71.4)	PRJEB15375	(*12*)
Australia 4	10	2	8	HCU	2	9 (90)	PRJEB15375	(*12*)
Denmark	4	0	4	HCU	1	4 (100)	PRJEB18427	(*13*)
New Zealand 1	2	0	2	HCU	1	2 (100)	PRJEB15375	(*12*)
New Zealand 2	3	0	3	HCU	1	3 (100)	PRJEB15375	(*12*)
New Zealand 3	5	0	5	HCU	1	5 (100)	PRJEB15375	(*12*)
New Zealand 4	2	0	2	HCU	1	2 (100)	PRJEB15375	(*12*)
United Kingdom	3	0	3	HCU	1	3 (100)	PRJNA324238	(*13*)
Zürich	2	0	2	HCU	2	1 (50)	PRJNA313770, PRJNA314007	(*9*)
Italy	1	1	0	Non-HCU	1	0	PRJEB18427	(*9*)
Maryland	1	1	0	Non-HCU	1	0	PRJNA345021	This study
Massachusetts 2	1	1	0	Non-HCU	1	0	PRJNA319839	This study
North Carolina	1	1	0	Non-HCU	1	0	PRJNA345021	This study
Pennsylvania 2	1	1	0	Non-HCU	1	0	PRJNA345021	This study
Tennessee	2	2	0	Non-HCU	1	0	PRJNA319839	This study
Texas	2	2	0	Non-HCU	1	0	PRJNA345021	This study
Total		38	90			112 (95)‡		

*US isolates were collected during 2015–2016. Status refers to HCU-associated isolates (HCU) collected directly from Stöckert 3T Heater–Cooler Units (LivaNova PLC, https://www.livanova.com; formerly Sorin Group Deutschland GmbH) or from patients with suspected HCU-derived *M. chimaera*, and isolates from pulmonary NTM patients without history of HCU exposure (non-HCU). HCU, heater–cooler unit; NCBI, National Center for Biotechnology Information. †Denotes the location from which the 2 samples (USA_PA_HCU_0, 2015–06–01; and USA_PA_PAT_10, 2015–22–65) did not pass the genomics quality control assessment and were excluded from the analyses.  ‡Percentage was derived from the number of HCU1 genotype isolates in the number of isolates collected directly from LivaNova 3T HCU or suspected patient cases.

**Figure 1:**
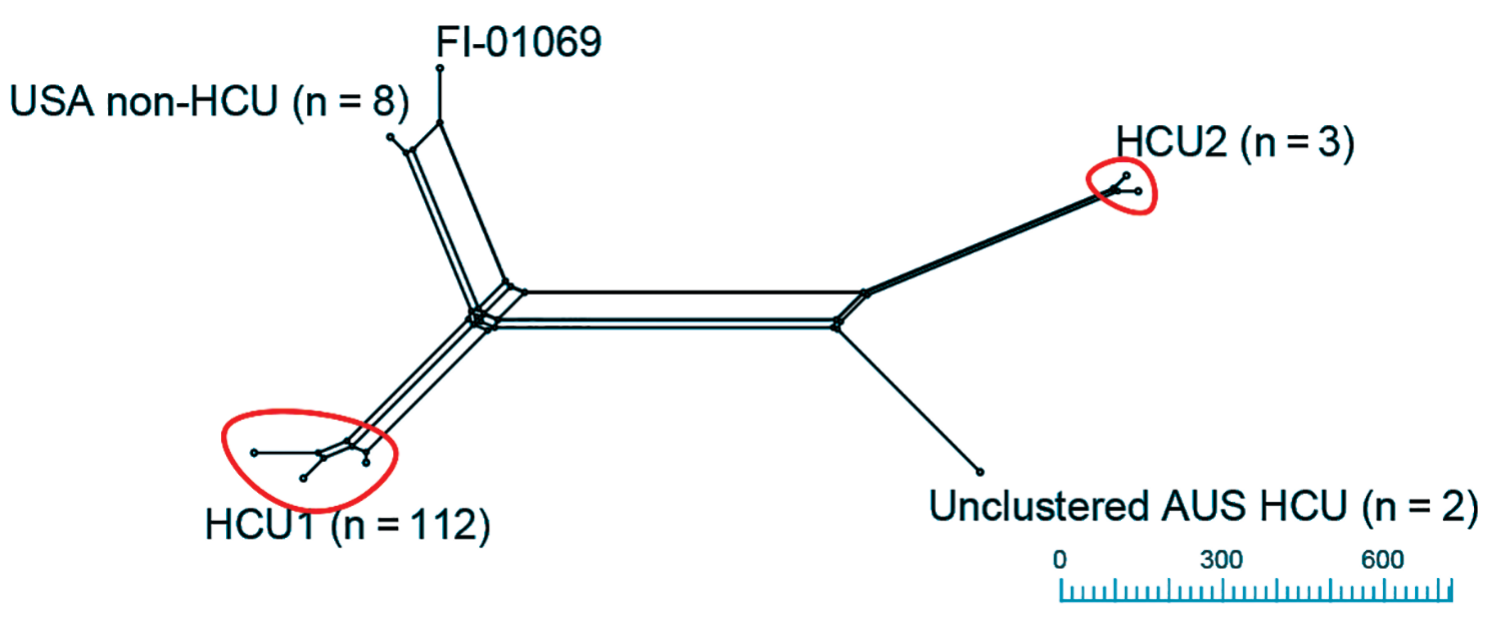
Neighbor Net splitstree of *Mycobacterium chimaera* isolates: relationships between *M. chimaera* isolates (n = 124) mapped against the *M. chimaera* strain CDC 2015–22–71 heater–cooler unit (HCU) reference genome (18,190 single nucleotide polymorphisms [SNPs] in 3,815,639 core positions). Isolates were grouped with a threshold of <500 SNPs to the nearest cluster. Clustered HCU isolates, including the reference strain CDC 2015-22-71, comprise the HCU1 cluster (n = 112) and HCU2 (n = 3). Unclustered isolates include Australian (AUS) HCU isolates (n = 2), USA non-HCU isolates (n=8), and the type strain FI-01069. Scale bar indicates SNPs.

**Figure 2:**
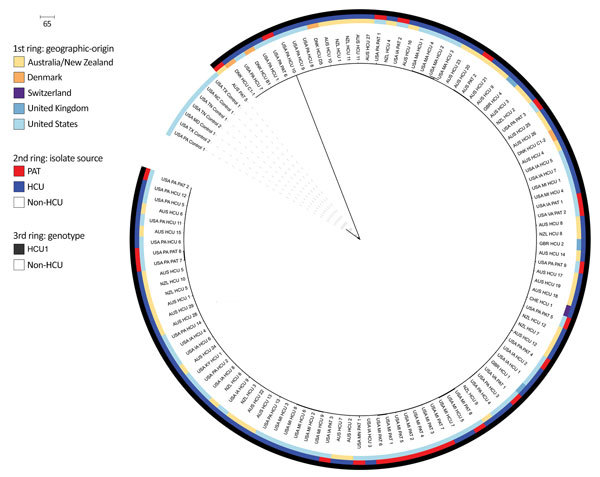
Maximum-likelihood phylogenetic relationships between HCU1 and US non-HCU–associated isolates (651 single-nucleotide polymorphisms [SNPs] in 4,024,718 core positions) as a circular phylogeny. From the center to the perimeter, colored circles indicate the country of origin, isolate source, and HCU genotype(s). Clinical isolate labels use country abbreviation: Australia (AUS), Denmark (DNK), Florence, Italy (FI), New Zealand (NZL), Switzerland (CHE), United Kingdom (GBR), United States (USA); HCU or PAT; isolate number. Non-HCU–associated isolates are from respiratory patients without a history of cardiac surgery. Suspected cases are isolates from blood or tissue samples collected from post–cardiac surgery patients; HCU are isolates collected from hospital HCUs (swabs, water, bioaerosols). Scale bar indicates SNPs. HCU, heater–cooler unit; PAT, suspected case-patient.

The NeighborNet splitstree (Appendix) of *M. chimaera* showed 3 groups (HCU1, HCU2, and non-HCU; Figure 1). Clade HCU1 (n = 112 isolates; Figure 2) is a discrete cluster composed entirely of HCU-associated isolates from case-patients and HCUs (mean pairwise distance 4 SNPs, range 0–23 SNPs; Appendix Figure 2) from Australia, Denmark, New Zealand, Switzerland, the United Kingdom, and the United States. Clade HCU2 was composed of 3 HCU-associated *M. chimaera* isolates from Switzerland (*2*) and Australia (AUS_HCU_30 and AUS_HCU_31). The mean difference among HCU2 isolates was 21.3 SNPs (range 19–25 SNPs; Appendix). Clade 3 was composed of US non–HCU-associated isolates (non-HCU). Two HCU-associated patient isolates from Australia (mean non–HCU-associated isolate pairwise SNPs 52, range 3–111 SNPs; Appendix) were unclustered. The mean distance between HCU1 and international HCU isolates was 13.58 SNPs (range 0–521 SNPs; Appendix Figure 3); the mean distance between HCU1 and non–HCU-associated isolates was 510.5 SNPs (range 506–610 SNPs; Appendix). In comparison, the mean distance between HCU2 and non-HCU isolates was 17,130.7 SNPs (range 17,057–17,221 SNPs). Of the 117 HCU-associated isolates we analyzed, 112 (95.7%) were HCU1 cluster, 3 (2.6%) were HCU2 cluster, and 2 isolates (1.7%) were not in a major clade.

Whole-genome sequencing of US HCU-associated *M. chimaera* isolates and their comparisons with global HCU-associated isolates provides further evidence for point-source contamination and worldwide dissemination of a *M. chimaera* strain (*3*–*5*). Twenty-two of 24 (92%) US patient isolates associated with HCU exposure during cardiac surgery phylogenetically clustered with international HCU-derived and post–cardiac surgery patient isolates, including those from Australia, Europe, and New Zealand (HCU1). None of the 8 US non–HCU-associated isolates were genetically similar to the HCU1 or HCU2 clusters. Isolates from US post–cardiac surgery patients were genetically more similar to isolates derived from international LivaNova 3T HCUs (mean pairwise distance 4 SNPs) than *M. chimaera* isolates from US patients without a history of cardiac surgery (mean pairwise distance 511 SNPs). This evidence supports the hypothesis that US post–cardiac surgery *M. chimaera* infections were acquired from exposure to factory-contaminated HCUs rather than local populations of waterborne *M. chimaera* in each hospital.

Our analyses revealed that all US *M. chimaera* isolates associated with LivaNova 3T HCU exposure genetically cluster with HCU1 genotype isolates implicated in the global outbreak of post–cardiac surgery *M. chimaera* infections. The HCU2 cluster was not observed in the United States but included 2 isolates from HCUs in Australia, as well as a representative genotype of *M. chimaera* found in HCUs in Europe and at the HCU production site. Consistent with previous findings, this finding suggests the international circulation of a second, less plentiful, strain in the manufacturing site water system (*8*).

These observations support the hypothesis that the LivaNova 3T HCU design provided suitable conditions for both NTM colonization and aerosolization, particularly by *M. chimaera*. Even though production site contamination with *M. chimaera* has been confirmed, the medical community needs to remain alert for HCU-associated NTM infections involving other species (*4*). HCUs are vulnerable to contamination from in-hospital water sources, use of improper water sources, and improper maintenance, each of which may increase the risk of infection by NTM (including *M. abscessus*, *M. chelonae*, and *M. gordonae*, in addition to *M. chimaera*) (*6*). Contaminated HCUs may contain NTM-contaminated biofilms. Furthermore, water from the LivaNova 3T HCUs can become aerosolized during normal function, leading to introduction of potentially infectious particles into the sterile field, onto graft materials, or into the open chest cavity during cardiac surgery. The death rate for HCU-associated *M. chimaera* infections has been reported to be 50%; the latent period to diagnosis can be up to 5 years postsurgery (*4*,*6*,*7*,*9*,*10*), further emphasizing necessary diligence on the part of physicians and cardiac surgery patients to monitor for symptoms of disseminated NTM infection.

Our study has some limitations in methodology. We did not obtain samples from every US hospital that reported LivaNova 3T HCU–associated *M. chimaera* cases; no submitting hospital collected all 3 types of samples (HCUs, non-HCU samples, and suspected case-patients); and HCU samples were not collected by a single person or according to a standardized collection protocol. Despite these limitations, this analysis of US HCU-associated *M. chimaera* isolates clearly shows the clustering of isolates from epidemiologically linked US cases to international LivaNova 3T HCU *M. chimaera* isolates and the HCU1 genotype found within the LivaNova manufacturing site.

## Conclusion

In conclusion, the application of WGS has advanced our understanding of *M. chimaera* present in US LivaNova 3T HCUs and patient cases after the initial analysis of suspected cases in Pennsylvania and Iowa. Given the innate drug resistance and the high death rate of HCU-associated *M. chimaera* infections, it remains imperative for hospitals to follow Food and Drug Administration guidelines (*9*) and the manufacturer’s instructions to minimize the risk of patient infection. In addition, clinicians should monitor patients who have had cardiac surgery using LivaNova 3T HCUs for signs and symptoms of NTM infection to enable early diagnosis and treatment.

**Appendix.** Details about genomic analysis of cardiac surgery–associated *Mycobacterium chimaera* infections, United States.
