# Supplementary material for: Genomic Analysis of Cardiac Surgery–Associated Mycobacterium chimaera Infections, United States
Source: Emerg Infect Dis. 2019 Mar;25(3):559–63. doi: 10.3201/eid2503.181282 (PMC6390774; doi:10.3201/eid2503.181282)
Supplement: Supplementary file 1 — Appendix. Details about genomic analysis of cardiac surgery–associated Mycobacterium chimaera infections, United States. [file 18-1282-Techapp-s1.pdf]

Article DOI: https://doi.org/10.3201/eid2503.181282

Genomic Analysis of Cardiac Surgery–Associated *Mycobacterium chimaera* Infections, United States

Appendix

Whole Genome Sequencing

In 2015 and 2016, nontuberculous mycobacterium (NTM) isolates from heater–cooler unit (HCU) water, HCU bioaerosols, and suspected patient cases were collected from 8 US locations (Table). These isolates were sequenced at National Jewish Health and the US Centers for Disease Control and Prevention (CDC). For isolates sequenced at CDC, species identification was performed by matrix-assisted laser desorption/ionization-time of flight mass spectrometry (MALDI-TOF MS) (Bruker Daltonics Inc., https://www.bruker.com/) with minimum scores of 2.0. Confirmed colonies were subcultured in 7H9 broth (Becton Dickinson, http://www.bd.com) at 37°C with 5% CO_2 until turbid. We extracted mycobacterial DNA from the 7H9 broth using the Maxwell 16 MDX (Promega, https://www.promega.com/) instrument and the Maxwell 16-cell LEV DNA purification kit. We constructed high-quality DNA libraries using NuGEN (https://www.nugen.com/) Ovation Ultralow library prep kits and sequenced using a 500-cycle Illumina (https://www.illumina.com) MiSeq reagent kit v2 for 2 × 250 bp paired-end sequence reads. For isolates sequenced at National Jewish Health (Denver, CO), we extracted mycobacterial DNA using a modified protocol (*1*), employing a column DNA clean in lieu of a phenol chloroform and ethanol precipitation. We determined species identification of HCU isolates through the amplification and sequencing of a 723 bp segment of the RNA polymerase β subunit (rpoB) gene, also known as region 5 (*2*). We trimmed sequences for quality and compared them against rpoB sequences deposited in the National Center for Biotechnology Information (NCBI) GenBank using the BLAST algorithm (https://blast.ncbi.nlm.nih.gov/Blast). Definitions of species by rpoB were those of the Clinical Laboratory Standards Institute (*3*). The National Jewish Health Human Subject Institutional Review Board (HS no. 2674) approved sequencing of NTM strains derived from patients. We constructed DNA libraries using Nextera XT (Illumina)

library prep kits, normalized manually for pooling, and sequenced using a 600-cycle Illumina MiSeq reagent kit v3 for 2 × 300 bp paired-end sequence reads.

Publicly Available *M. chimaera* Genome Retrieval

We downloaded a subset of sequence reads corresponding to publicly available *M. chimaera* genomes from isolates collected in Australia and New Zealand (*4,5*), Denmark (*6*), Italy (*7*), the United Kingdom (*8*), and Switzerland (*7*) from the NCBI Sequence Read Archive (SRA) using the SRA toolkit (*9*). These are listed in the Table in the main article.

Phylogenomic Analyses

Sequence reads were trimmed of sequencing adapters and with a quality Phred score threshold of 30 (*10*) and mapped to the *M. chimaera* strain CDC 2015–22–71 reference genome (GenBank accession no. NZ_CP019221.1) using the Single Nucleotide Variant Phylogenomics (SNVPhyl) pipeline for paired-end Illumina data (*11*). The paired-end reads were aligned to the reference genome to generate read pileups (SMALT v.0.7.5; 5; http://www.sanger.ac.uk/science/tools/smalt-0) followed by quality filtering of single nucleotide polymorphisms (SNPs) and coverage estimations. From each pileup, the variant calling, variant consolidation and SNP alignment generation of the final phylogeny was run through PhyML (*12*) using maximum likelihood. Only isolates that had a minimum of 80% of the reference genome, at 20× or greater coverage, and/or had less than 10% undetermined bases (N) or insertions/deletions (indels) introduced during SNP calling were included in subsequent analyses. The resulting phylogenetic tree was visualized using Splitstree5 (*13*) and the R package GGTREE (*14*).

Genomes sequenced in this project are available online on the NCBI network under accession PRJNA345021 (http://www.ncbi.nlm.nih.gov/bioproject/345021).

References

1. Käser M, Ruf M-T, Hauser J, Marsollier L, Pluschke G. Optimized method for preparation of DNA from pathogenic and environmental mycobacteria. Appl Environ Microbiol. 2009;75:414–8. http://dx.doi.org/10.1128/AEM.01358-08

2. Ben Salah I, Adékambi T, Raoult D, Drancourt M. rpoB sequence-based identification of *Mycobacterium avium* complex species. Microbiology. 2008;154:3715–23. http://dx.doi.org/10.1099/mic.0.2008/020164-0

3. Clinical and Laboratory Standards Institute. Interpretive criteria for identification of bacteria and fungi by DNA target sequencing. 1st ed. CLSI guideline MM18-A. Wayne, PA: The Institute, 2008.

4. Robinson JO, Coombs GW, Speers DJ, Keehner T, Keil AD, D'Abrera V, et al. *Mycobacterium chimaera* colonisation of heater-cooler units (HCU) in Western Australia, 2015: investigation of possible iatrogenic infection using whole genome sequencing. Euro Surveill. 2016;21:30396. http://dx.doi.org/10.2807/1560-7917.ES.2016.21.46.30396

5. Williamson D, Howden B, Stinear T. *Mycobacterium chimaera* spread from heating and cooling units in heart surgery. N Engl J Med. 2017;376:600–2. http://dx.doi.org/10.1056/NEJMc1612023

6. Svensson E, Jensen ET, Rasmussen EM, Folkvardsen DB, Norman A, Lillebaek T. *Mycobacterium chimaera* in heater-cooler units in Denmark related to isolates from the United States and United Kingdom. Emerg Infect Dis. 2017;23:507–9. http://dx.doi.org/10.3201/eid2303.161941

7. van Ingen J, Kohl TA, Kranzer K, Hasse B, Keller PM, Katarzyna Szafrańska A, et al. Global outbreak of severe *Mycobacterium chimaera* disease after cardiac surgery: a molecular epidemiological study. Lancet Infect Dis. 2017;17:1033–41. http://dx.doi.org/10.1016/S1473-3099(17)30324-9

8. Chand M, Lamagni T, Kranzer K, Hedge J, Moore G, Parks S, et al. Insidious risk of severe *Mycobacterium chimaera* infection in cardiac surgery patients. Clin Infect Dis. 2017;64:335–42. http://dx.doi.org/10.1093/cid/ciw754

9. Leinonen R, Sugawara H, Shumway M, International Nucleotide Sequence Database Collaboration. The sequence read archive. Nucleic Acids Res. 2010;39(suppl1):D19–D21. http://dx.doi.org/10.1093/nar/gkq1019

10. Jiang H, Lei R, Ding S-W, Zhu S. Skewer: a fast and accurate adapter trimmer for next-generation sequencing paired-end reads. BMC Bioinformatics. 2014;15:182. http://dx.doi.org/10.1186/1471-2105-15-182

11. Petkau A, Mabon P, Sieffert C, Knox NC, Cabral J, Iskander M, et al. SNVPhyl: a single nucleotide variant phylogenomics pipeline for microbial genomic epidemiology. Microb Genom. 2017;3:e000116. http://dx.doi.org/10.1099/mgen.0.000116

12. Guindon S, Dufayard JF, Lefort V, Anisimova M, Hordijk W, Gascuel O. New algorithms and methods to estimate maximum-likelihood phylogenies: assessing the performance of PhyML 3.0. Syst Biol. 2010;59:307–21. http://dx.doi.org/10.1093/sysbio/syq010

13. Huson DH, Bryant D. Application of phylogenetic networks in evolutionary studies. Mol Biol Evol. 2006;23:254–67. http://dx.doi.org/10.1093/molbev/msj030

14. Yu G, Smith DK, Zhu H, Guan Y, Lam TTY. GGTREE: an R package for visualization and annotation of phylogenetic trees with their covariates and other associated data. Methods Ecol Evol. 2017;8:28–36. http://dx.doi.org/10.1111/2041-210X.12628

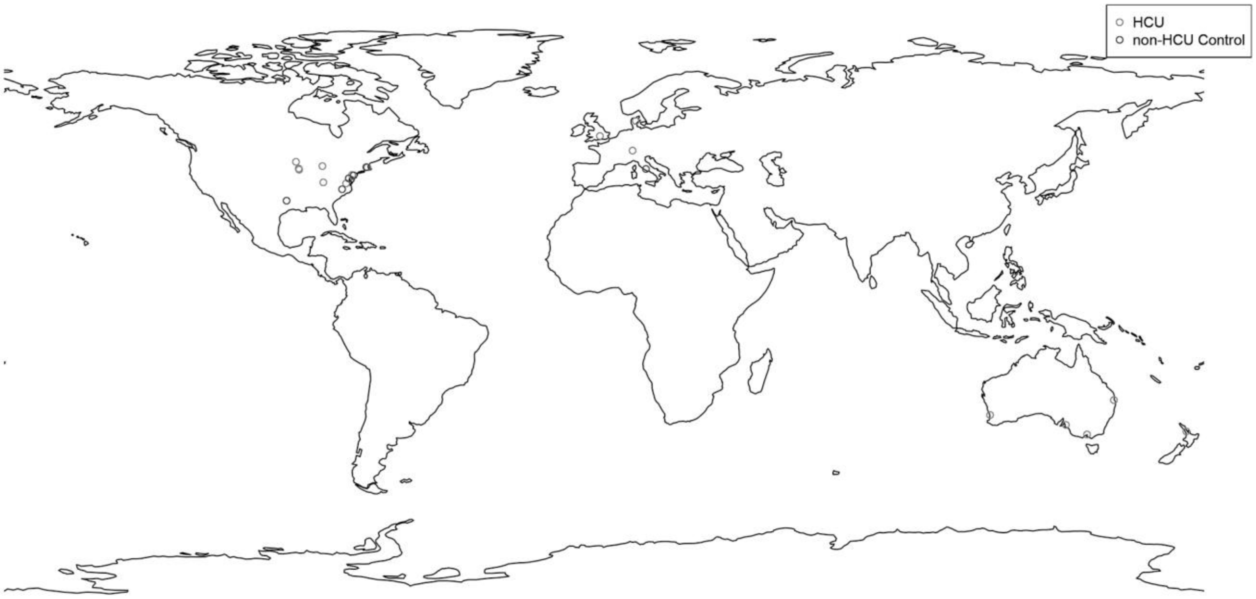

Appendix Figure 1. Global locations of heater–cooler unit (HCU), suspected HCU patient case, and non-HCU–associated *Mycobacterium chimaera* isolate genomes included in this study. *M. chimaera* isolates from 13 US locations were collected for whole genome sequencing and compared against available HCU and non-HCU *M. chimaera* genomes from Australia, Denmark, New Zealand, Switzerland, and the United Kingdom.

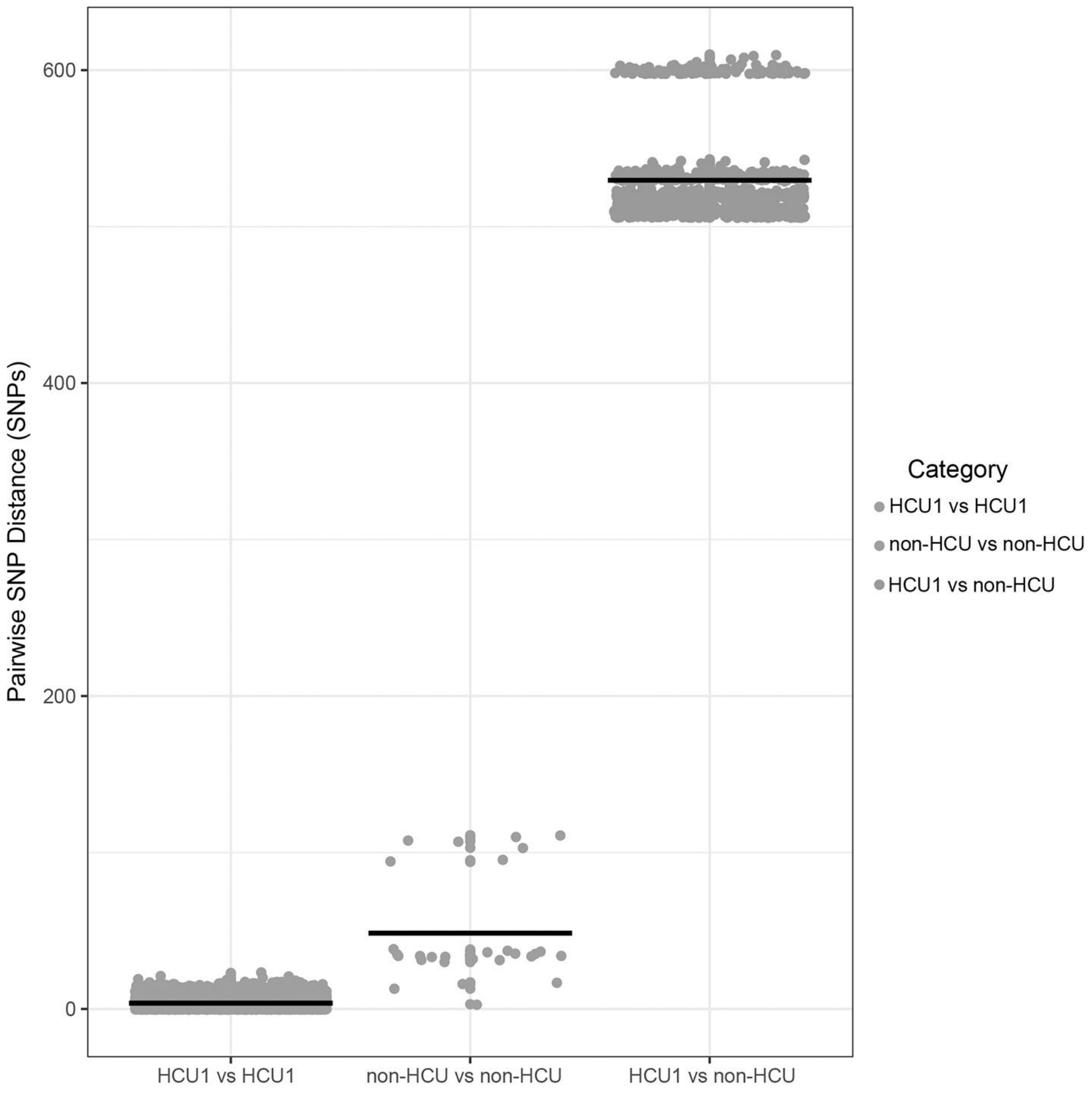

Appendix Figure 2. Scatterplot of pairwise single nucleotide polymorphisms (SNPs) between HCU1 and non-HCU–associated *Mycobacterium chimaera* isolates. SNPs were observed in the alignment of 4,024,718 core nucleotide positions (no Ns or indels) and categorized by the groups of genomes compared (HCU1 versus non-HCU isolates). Black line represents the mean SNP differences observed per categorical comparison.

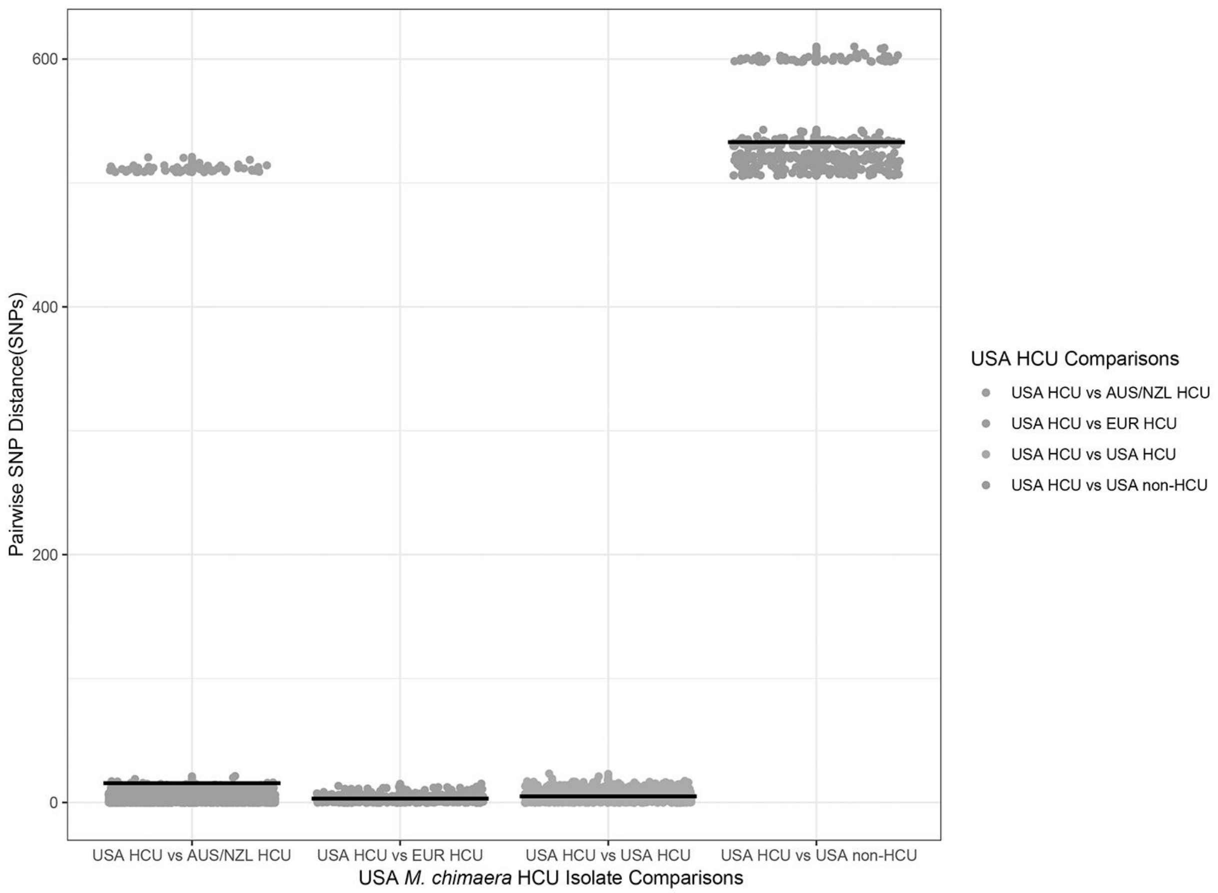

Appendix Figure 3. Scatterplot of pairwise single nucleotide polymorphisms (SNPs) between US HCU1, global HCU, and US non-HCU–associated *Mycobacterium chimaera* isolates. SNPs were observed in the alignment of 18,190 SNPs in 3,815,639 core positions positions (no Ns or indels) and categorized by the groups of genomes compared (HCU1 versus non-HCU isolates). Black line represents the mean SNP differences observed per categorical comparison.
